# LARP7 overexpression alleviates aortic senescence and atherosclerosis

**DOI:** 10.1111/jcmm.18388

**Published:** 2024-05-31

**Authors:** Ping Yang, Shuo Wu, Yige Li, Yingmei Lou, Junhao Xiong, Yuze Wang, Zilong Geng, Bing Zhang

**Affiliations:** ^1^ Key Laboratory of Systems Biomedicine, Shanghai Center for Systems Biomedicine, Engineering Research Center of Techniques and Instruments for Diagnosis and Treatment of Congenital Heart Disease, Institute for Developmental and Regenerative Medicine, Xin Hua Hospital, School of Medicine Shanghai Jiao Tong University Shanghai China; ^2^ Key Laboratory of Systems Biomedicine, Shanghai Center for Systems Biomedicine, Department of Cardiovascular Surgery, Shanghai Chest Hospital, Engineering Research Center of Techniques and Instruments for Diagnosis and Treatment of Congenital Heart Disease, Institute for Developmental and Regenerative Medicine, Xin Hua Hospital, School of Medicine Shanghai Jiao Tong University Shanghai China

**Keywords:** atherosclerosis, cellular senescence, Larp7, Sirt1

## Abstract

Atherosclerosis, characterized by the accumulation of lipid plaques on the inner walls of arteries, is the leading cause of heart attack, stroke and severe ischemic injuries. Senescent cells have been found to accumulate within atherosclerotic lesions and contribute to the progression of atherosclerosis. In our previous study, we discovered that suppressing Larp7 accelerates senescence by inhibiting Sirt1 activity, resulting in increased atherosclerosis in high‐fat diet (HFD) fed and *ApoE* deficient (*ApoE* ^KO^) mice. However, there has been no direct evidence demonstrating Larp7 per se could attenuate atherosclerosis. To this end, we generated a tetO‐controlled and Cre‐activated Larp7 gain‐of‐function mouse. Through RT‐PCR and western blotting, we confirmed Larp7 overexpression in the aortas of HFD‐fed *ApoE* ^KO^; *Larp7* ^tetO^ mice. Larp7 overexpression led to increased Sirt1 activity and decreased cellular senescence signals mediated by p53/p65 in the aortas. Additionally, Larp7 overexpression reduced the presence of p16‐positive senescent cells in the aortic lesions. Furthermore, Larp7 overexpression resulted in a decrease in pro‐inflammatory macrophages and SASP factors. Consequently, Larp7 overexpression led to a reduction in the area of atherosclerotic lesions in HFD‐fed *ApoE* ^KO^; *Larp7* ^tetO^ mice. In summary, our study provides evidence that Larp7 overexpression holds promise as an approach to inhibit cellular senescence and prevent atherosclerosis.

## 1 INTRODUCTION

Atherosclerosis, a type of ageing‐related disease, is the leading cause of death in the industrialized world.^1^ Atherosclerosis is caused by a build‐up of plaques filled by oxidative lipid substances on the inner arterial walls. Overtime, plaques accumulated and get unstable, which is the leading cause of heart attack, stroke and severe ischemic injuries. Cellular senescence, a hallmarker of ageing, occurred in various cell types accompanied with a pro‐inflammatory phenotype, termed the senescence‐associated secretory phenotype (SASP), plays a dominant role in atherosclerosis.^2^, ^3^, ^4^ Removal of senescent cells or repress the pro‐inflammatory SASP exhibits a promising potential for treating atherosclerosis.^5^, ^6^, ^7^, ^8^

Sirtuins are a well‐known group of NAD + ‐dependent deacetylase and deacylase enzymes that play a crucial role in regulating lifespan and the ageing process.^9^, ^10^, ^11^ Sirtuins regulate crucial cellular processes such as DNA damage, cellular senescence, inflammation, mitochondrial function and cell metabolism, which are disrupted in ageing and cardiovascular disease.^11^, ^12^, ^13^, ^14^ Activating sirtuins or boosting NAD+ levels has been shown to decelerate key ageing processes and induce lipid‐lowering and anti‐inflammatory effects in various cardiovascular diseases, including atherosclerosis.^13^, ^15^, ^16^ Sirt1, a key member of the sirtuin family, was considered as the most promising therapeutic target in ageing and cardiovascular disease. Sirt1 safeguards against atherosclerosis by inhibiting endothelial cell dysfunction, senescence of vascular smooth muscle cells (VSMCs) and the formation of macrophage foam cells.^17^, ^18^, ^19^ This protective mechanism is achieved through the regulation of DNA damage repair, anti‐apoptotic pathways and anti‐inflammatory pathways.

Larp7, a member of the La family RNA binding proteins, is recognized for its involvement in regulating RNA polymerase II pausing and release through its binding to 7SK RNA.^20^ Our previous studies have shown that Larp7 serves as a novel activator of Sirt1 deacetylase and plays a protective role in age‐related disorders and cardiovascular diseases.^21^, ^22^ The protein levels of Larp7, but not its mRNA, were significantly decreased in the aorta, skin, intestine and other tissues of ageing mice.^21^ Persistent DNA damage in ageing mice triggers a DNA damage response (DDR) and activates the ataxia telangiectasia mutated (ATM) pathway, resulting in the subcellular mis‐localization and degradation of the Larp7 protein.^21^ The reduced levels of Larp7 protein result in a decline in Sirt1 deacetylase activity and an increase in p53/p65‐mediated cellular senescence.^21^ The ATM‐Larp7‐Sirt1‐p53/p65 axis plays a key role in vascular senescence and ageing, and inhibition of this axis with an ATM inhibitor has been shown to alleviate atherosclerosis.^21^ However, it is still unclear whether overexpressing Larp7 could be a targeted approach for treating atherosclerosis.

Here, to explore the therapeutic potential of Larp7 overexpression in treating atherosclerosis, we induced Larp7 overexpression in a *ApoE* knockout (*ApoE* ^KO^) mouse by utilizing Tet‐on system and Cre‐loxp system. Molecular and Histological analysis revealed Larp7 overexpression activated the Larp7‐Sirt1‐p53/p65 axis, resulting in alleviation of cellular senescence and atherogenesis in the *ApoE* ^KO^; *Larp7* ^tetO^ mouse. In conclusion, our study provides evidence that Larp7 overexpression is a promising approach for preventing ageing‐related vascular pathologies.

## 2 MATERIALS AND METHODS

### 2.1 Animals

*Larp7* ^tetO^ mouse was generated from the Shanghai Biomodel Organism Science & Technology Development Corporation (Shanghai, China) by using CRISPR‐Cas9 technology. To site‐specific knock‐in of the SA‐pA‐tetO‐LSL‐Larp7‐CAG‐M2rtTA expression cassette at the Col1a1 gene locus through homologous recombination, a donor vector containing a 3.0‐kb 5′ homology arm, a 10.8‐kb knock‐in fragment and a 2.9‐kb 3′ homology arm was constructed using In‐Fusion cloning. Two guide RNAs targeting Col1a1 gene were designed: gRNA1, 5′‐3′ GCCCCTTCTATACTAAATTA; gRNA2, 5′‐3′ AATGCTGGGGTGTCACAAGG. Cas9 mRNA, gRNA and the donor vector were microinjected into fertilized eggs of C57BL/6J mice to generate F0 mice. The correctly targeted F0 mice were bred with C57BL/6J mice to obtain positive F1 mice. Specific primers were employed for genotyping *Larp7* ^tetO^ mice: P1, 5′‐3′ TGCTCGCACGTACTTCATTC, P2, 5′‐3′ CATCAAGGAAACCCTGGACTAC.

UBC^CreERT2^ mice (129S.Cg‐*Ndor1* ^*Tg*(*UBC‐cre*/*ERT2*)*1Ejb*^/J, 007179)^23^ were purchased from the Jackson Laboratory. The inducible *Larp7* gain‐of‐function mice were generated by crossing *Larp7* ^tetO^ mice with UBC‐Cre^ERT2^ mice. *ApoE* ^KO^ mice (C57BL/6‐Apoe^em5Smoc^, NM‐KO‐190565) were purchased from the Shanghai Biomodel Organism Science & Technology Development Corporation (Shanghai, China).

To explore the role of *Larp7* overexpression in atherosclerosis, *Larp7* ^tetO^; UBC‐Cre^ERT2^ mice were crossed with *ApoE* ^KO^ hyperlipidaemic mice to generate *Larp7* overexpression hyperlipidaemic mice (*ApoE* ^KO^; *Larp7* ^tetO^). The mice of 6 to 8 weeks were randomly assigned to experimental groups and injected with tamoxifen (50 mg/kg mice, every other day for total of five times) intraperitoneally.^21^ To introduce the expression of Larp7, *ApoE* ^KO^; *Larp7* ^tetO^ mice were kept watering with 1 mg/mL doxycycline (Sigma, D9891)^24^ until the end of the experiment. *ApoE* ^KO^; *Larp7* ^tetO^ mice were fed with a western diet (Research Diets, D12108C) with 40% fat and 1.25% cholesterol for 12 weeks before being sacrificed. *ApoE* ^KO^; WT mice administered with a vehicle instead of doxycycline were used as a control. The mice were housed under standard conditions with a 12‐h light/dark cycle and had free access to water and standard chow unless specified. All animal protocol and procedures were approved by the Institute Animal Care and Use Committee (IACUC) of Shanghai Jiao Tong University.

### 2.2 RT‐qPCR

Total RNA was extracted from isolated aorta by using RNAsimple Total RNA Kit (TIANGEN Biotech) according to the manufacturer's instruction. HiScript II Q RT SuperMix with gDNA wiper (Vazyme, China) and quantified by the real‐time PCR using ChamQ Universal SYBR qPCR Master Mix kit (Vazyme, China) in Roche instruments. The following primers were used as follows: 5′‐GCCATTGAGTTTCTGAACAACC‐3′ and 5′‐CTTCGTGGCTTTACACACGC‐3′ for mouse *Larp7*; 5′‐ACGTGGCCTTGTCGCTGTCT‐3′ and 5′‐GACCAATCTGCGCTTGGAGTG‐3′ for mouse *Cdkn1a*; 5′‐ GCAACTGTTCCTGAACTCAACT‐3′ and 5′‐ ATCTTTTGGGGTCCGTCAACT‐3′ for mouse *Il1b*; 5′‐ TAGTCCTTCCTACCCCAATTTCC‐3′ and 5′‐ TTGGTCCTTAGCCACTCCTTC‐3′ for mouse *Il6*; 5′‐ TTAAAAACCTGGATCGGAACCAA‐3′ and 5′‐ GCATTAGCTTCAGATTTACGGGT‐3′ for mouse *Ccl2*; 5′‐ GTGATGCTCAGGTATCCATCCA‐3′ and 5′‐ CACAGTTCTCAAAGCACAGCG‐3′ for mouse *Icam1*; 5′‐ AGTTGGGGATTCGGTTGTTCT‐3′ and 5′‐ CCCCTCATTCCTTACCACCC‐3′ for mouse *Vcam1*; 5′‐ ACATGGAGACTTTGTCCCTTTTG‐3′ and 5′‐ TTGGCTGAGTGGTAGAGTCCC‐3′ for mouse *Mmp3*; 5′‐ CTGGACAGCCAGACACTAAAG‐3′ and 5′‐ CTCGCGGCAAGTCTTCAGAG‐3′ for mouse *Mmp9*; 5′‐ AAGAGCTATGAGCTGCCTGA‐3′ and 5′‐ TACGGATGTCAACGTCACAC‐3′ for mouse β‐actin.

### 2.3 Western blotting

Isolated aortas were lysed in high‐salt buffer B (20 mM HEPES pH 7.9, 450 mM NaCl, 25% Glycerol, 0.2 mM EDTA, 0.5 mM DTT, 1% NP40, 0.5% SDS) with protease inhibitors (Roche) for 20 min on ice. The isolated proteins were separated on SDS‐PAGE gels and transferred to 0.45 μm PVDF membrane. The membrane was blocked with 5% BSA in TBS/0.1% Tween 20 (TBST) and then incubated with primary antibodies: rabbit anti‐LARP7 antibody (1:1000, BETHYL, A303‐72A), or rabbit anti‐NF‐κB p65 antibody (1:1000, CST, 8242), or rabbit anti‐acetyl‐NF‐κB p65 (Lys310) antibody (1:1000, CST, 3045), or mouse anti‐p53 antibody (1:1000, Santa Cruz Biotechnology, sc‐126), or rabbit anti‐acetyl‐p53 (Lys382) antibody (1:250, Thermo Fisher, 710294), or rabbit anti‐p16 antibody (1:1000, Sigma‐Aldrich, SAB4500072), or rabbit anti‐β‐Tubulin antibody (1:1000, CST, 2146) overnight at 4°C. Horseradish peroxidase‐conjugated goat anti‐rabbit or anti‐mouse IgG served as secondary antibodies (1:3000, Cell Signaling Technology). The blots were imaged within Amersham Imager 600 after developing with an enhanced chemiluminescence substrate (Immobilon Western, WBKLS0500, Millipore).

### 2.4 SIRT1 activity assay

Our previous study has demonstrated that Larp7 specifically activates Sirt1, rather than other sirtuins. To assess the changes in Sirt1 activity following Larp7 overexpression, we utilized a Universal SIRT Activity Assay Kit (ab156915; Abcam) to measure nuclear SIRT activity. In brief, aortas were initially homogenized in buffer A (10 mM HEPES pH 7.9, 10 mM KCl, 1.5 mM MgCl2, 0.05% NP40 and 0.5 mM DTT) to remove cytoplasmic proteins and then lysed with buffer B (20 mM HEPES pH 7.9, 450 mM NaCl, 0.5 mM DTT). Following quantification using BCA assays, an equal amount of extract was directly used for the SIRT activity assay as per the manufacturer's instructions. The absorbance at 450 nm was measured using a BioTeK microplate reader. The SIRT activity was calculated using the following formula:

$$\text{SIRT activity}\;\left(\mathrm{OD}/\min/\mathrm{mg}\right)=\left(\frac{\text{Sample}\;\mathrm{OD}-\mathrm{NNC}\;\mathrm{OD}}{\text{Protein amount}\;\left(\mathrm{\mu g}\right)\times \text{Incubation time}\;\left(\min\right)}\right)\times 1000$$

here, NNC refers to control wells that do not contain the SIRT co‐factor NAD^+^.

### 2.5 Oil Red O staining

To measure atherosclerotic lesions in *ApoE* ^KO^; *Larp7* ^tetO^ mice and *ApoE* ^KO^; WT mice after HFD feeding, we isolated mice aortas and performed Oil Red O staining. To perform en face Oil Red O staining, mice aortas were dissected in cold PBS and fixed in 4% paraformaldehyde at 4°C for 24 h. For the Oil Red O staining of aortic roots, aortas were isolated and freshly embedded in O.C.T. compounds (Tissue‐Tek), 8 μm cryosections were fixed in 4% paraformaldehyde at room temperature for 15 min. To begin the staining process, the samples were first rinsed with water for 5 min, followed by a 5‐min rinse with 60% isopropanol. The aortas and aortic roots were then stained with 0.2% Oil Red O (Sigma, O0625) for 1 h at room temperature with gentle shaking. After staining, they were rinsed once more with 60% isopropanol for 5 min and then rinsed three times with water. The stained aorta en face was carefully opened, fixed on a black plate with the endothelium facing upwards and captured using a Nikon DS‐Ri2 stereomicroscope. The image of the aortic root section was captured with a Nikon & NI‐U upright microscope. The lesion area was quantified using the ImageJ package. All quantifications of the aorta en face and aortic root were performed by experimenters who were blinded to the group design.

### 2.6 Immunofluorescence

Aortas cryostat sections from −80°C were air‐dried at room temperature for 10 min, fixed in a pre‐cold mixture of methanol and acetone (1:1) for 10 min at −20°C, air‐dried for 10 min and then rehydrated in PBS for 5 min. Antigen retrieval was performed by incubating the sections in citrate retrieval buffer (10 mM sodium citrate, pH 6.0) at 95°C for 20 min. After blocking with a blocking buffer (5% normal donkey serum, 1% BSA and 0.3% Triton X‐100 in PBS) for 30 min at room temperature, the sections were incubated overnight at 4°C with rabbit anti‐p16 (1:200; Abcam, ab54210) or Rat anti‐Mac3 primary antibodies (1:200, BD Bioscience, 553322) in a dilution buffer (5% normal donkey serum, 1% BSA and 0.1% Triton X‐100 in PBS). Subsequently, the sections were incubated with Alexa Fluor 488‐ or 555‐conjugated secondary antibodies (1:500; Thermo Fisher Scientific) for 1 h at room temperature. Nuclear staining was performed using Hoechst 33342 (Thermo Fisher Scientific). Fluorescent images were captured with the Nikon A1Si confocal microscope. The average fluorescent intensity of p16 and Mac3 was quantified using the ImageJ package through a double‐blind method.

### 2.7 Blood lipid measurement

To evaluate the effects of LARP7 overexpression on blood lipid, blood samples were collected from *ApoE* ^KO^; *Larp7* ^tetO^ mice and *ApoE* ^KO^; WT mice after HFD feeding. Plasmas were harvested by centrifuging for 10 min at 1500 rpm. Total plasmatic cholesterol and triglyceride were measured using Total Cholesterol Quantification Kit (A111‐1‐1, Nanjing Jiancheng Bioengineering Institute) and Triglyceride Quantification Kit (A110‐1, Nanjing Jiancheng Bioengineering Institute), respectively, according to the manufacturer's instructions.

### 2.8 Statistics

Each assay was repeated at least three times independently. The data were presented as Mean ± Standard Deviation (SD). Comparisons between two groups were analysed by two‐tailed parametric *t*‐test if the data were normally distributed, otherwise, the Mann–Whitney *U*‐test was employed. Statistical analysis was performed by GraphPad Prism 9. The detailed biological replicates in each group were indicated in figure legends. A difference was considered significant if *p* < 0.05.

## 3 RESULTS

### 3.1 Generating a tetO‐controlled and Cre‐activated Larp7 overexpression mouse

**FIGURE 1:**
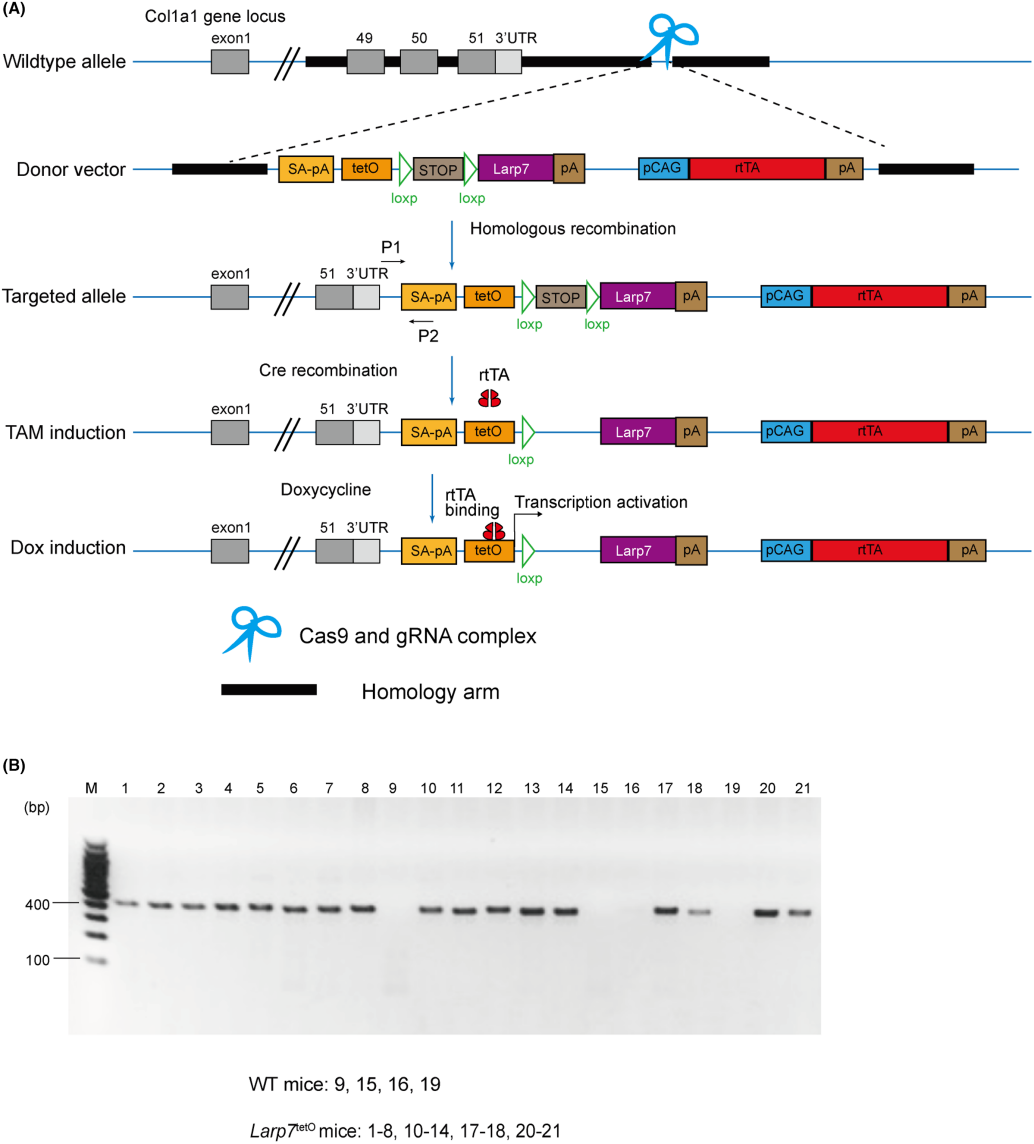
Strategy for generating inducible *Larp7* ^tetO^ mouse. (A) Strategy for generating an inducible overexpression mouse by combining the Tet‐On system with the Cre‐loxp system. SA‐pA means splice acceptor and polyA signal. P1 and P2 are specific primers for *Larp7* ^tetO^ mice genotyping. (B) Representative gel image of genotyping results. *Larp7* ^tetO^ mice had a 413 bp product band.

To explore the therapeutic potential of Larp7 overexpression in treating atherosclerosis, we first generated a tetO‐controlled and Cre‐activated Larp7 gain‐of‐function mouse (named as *Larp7* ^tetO^, Figure 1A) by using CIRSPR‐Cas9‐mediated homology recombination (HR). Cas9 protein and guide RNAs induced a double‐strand break at the Col1a1 gene loci; a co‐transfected donor vector with two homology arms was inserted into these broken loci. The donor vector in this study contained two open reading frames (ORFs): one ORF consisted of a tetracycline‐responsive promoter (P_tet_) with tetO sequences, a premature stop codon flanked by a pair of loxp sites in the same direction (abbreviated as LSL), *Larp7* coding sequences (CDS) and a polyA signal; the other ORF contained a CAG promoter, reverse‐tTA (rtTA) variant M2 coding sequences and a second polyA signal.

*Larp7* ^tetO^ mice firstly crossed with UBC‐Cre^ERT2+^ mouse to obtain tamoxifen‐inducible nuclear Cre expression elements, which allowed for the removal of the premature stop codon upstream of the *Larp7* CDS sequence. Subsequently, UBC‐Cre^ERT2+^; *Larp7* ^tetO^ mice were crossed with *ApoE* ^KO^ mice to generate *ApoE* ^KO^; *Larp7* ^tetO^ mice. Administration of doxycycline in *ApoE* ^KO^; *Larp7* ^tetO^ mice triggered a conformational switch in rtTA, enabling binding to tetO. The subsequent activation of the Ptet promoter led to the expression of the downstream *Larp7* gene. The combination of Tet‐on system and Cre‐loxp system allowed for time‐controllable expression of Larp7 in various tissues. Using allele‐specific primers, we confirmed the successful inheritance of the *Larp7* overexpression elements in *Larp7* ^tetO^ offspring (Figure 1B).

### 3.2 Larp7 overexpression in *Apo^EKO^*; *Larp7* ^*tetO*^ mouse boostered Sirt1 activity

**FIGURE 2:**
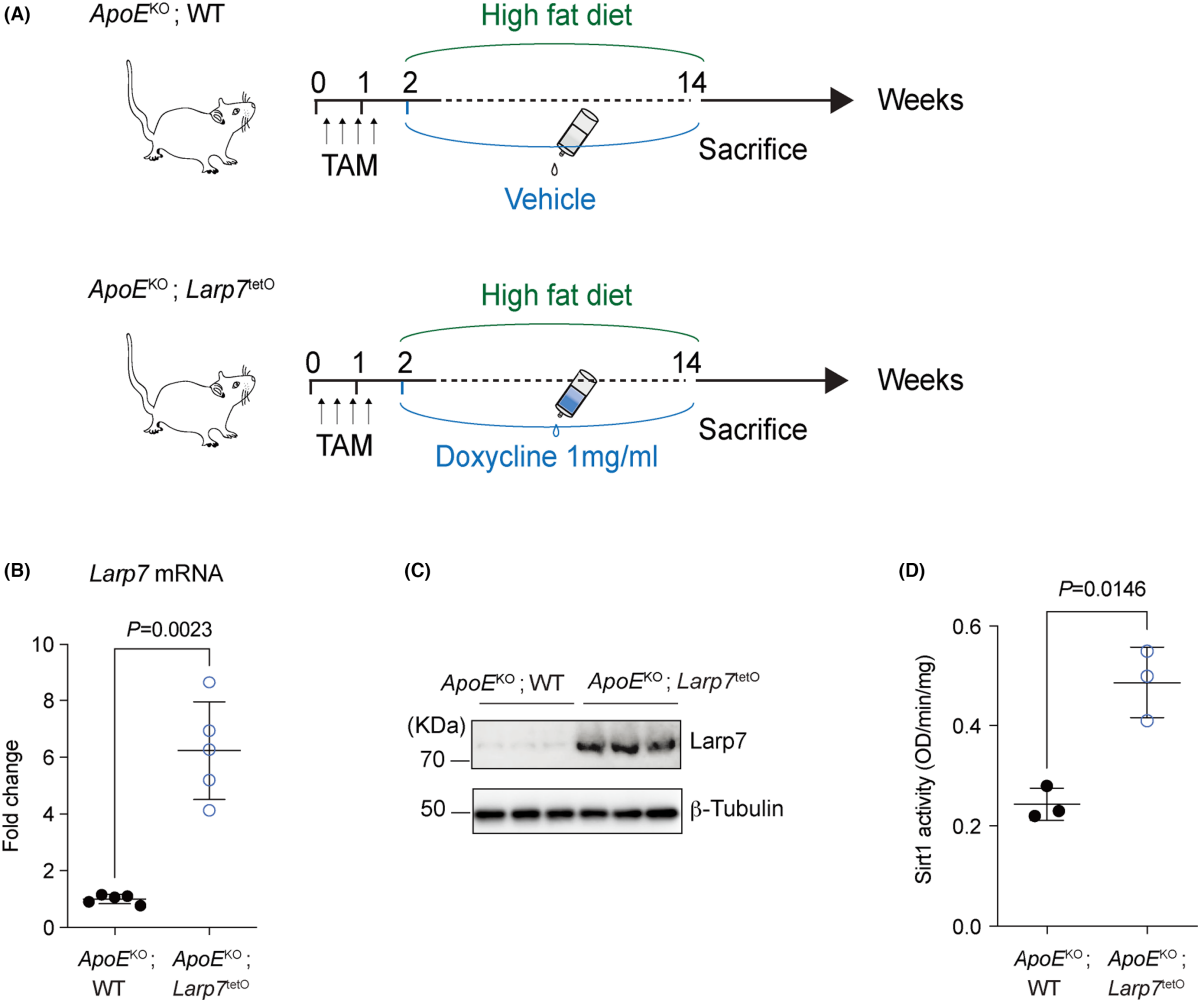
Larp7 overexpressed in *ApoE* ^KO^; *Larp7* ^tetO^ mice and activated Sirt1 in the arotas. (A) Experimental schedule. Tamoxifen (TAM) was consecutively injected at 5‐day intervals, followed by a 12‐week high‐fat diet (HFD) to expedite the progression of atherosclerosis. During the HFD period, oral administration of doxycycline was utilized to induce Larp7 overexpression in *ApoE* ^KO^; *Larp7* ^tetO^ mice. *ApoE* ^KO^; WT mice treated with a vehicle were used as controls. (B) Larp7 mRNA overexpressed in aortas of *ApoE* ^KO^; *Larp7* ^tetO^ mice compared with *ApoE* ^KO^; WT mice. *n* = 5 mice per group. Data = Mean ± SD, two‐tailed student's *t*‐test. *p* < 0.05 indicated significance. (C) Larp7 protein increased in the aortas of *ApoE* ^KO^; *Larp7* ^tetO^ mice compared with *ApoE* ^KO^; WT mice. (D) Sirt1 activity assays illustrating nuclear Sirt1 activity significantly increased in aortas of *ApoE* ^KO^; *Larp7* ^tetO^ mice compared with *ApoE* ^KO^; WT mice. *n* = 3 mice per group. Data = Mean ± SD, two‐tailed student's *t*‐test. *p* < 0.05 indicated significance.

To induce overexpression of Larp7, *ApoE* ^KO^; *Larp7* ^tetO^ mice were intraperitoneally injected with tamoxifen (TAM) consecutively for 5 days, followed by oral administration of 1 mg/mL doxycycline over a period of 12 weeks (Figure 2A). As a control, *ApoE* ^KO^; WT mice were treated with a vehicle instead of doxycycline oral administration. Both *ApoE* ^KO^; *Larp7* ^tetO^ mice and *ApoE* ^KO^; WT mice were fed a high‐fat diet (HFD) for 12 weeks to accelerate the development of atherosclerosis.

After induction with TAM injection and doxycycline oral administration, we observed over 6‐fold increase in *Larp7* mRNA (6.24 ± 1.72‐fold, *p* = 0.0023) in the aortas of *ApoE* ^KO^; *Larp7* ^tetO^ mice compared with *ApoE* ^KO^; WT mice (Figure 2B). Western blotting also demonstrated a significant elevation of Larp7 protein levels in *ApoE* ^KO^; *Larp7* ^tetO^ mice after 12 weeks of high‐fat diet (Figure 2C). These findings confirm the successful overexpression of Larp7 in *ApoE* ^KO^; *Larp7* ^tetO^ mice.

Our previous studies have established Larp7 as a new activator of Sirt1, and the reduced Sirt1 activity has been implicated in atherosclerosis. Therefore, we further examined the nuclear activity of Sirt1 in the aortas of HFD‐fed *ApoE* ^KO^; *Larp7* ^tetO^ mice. Remarkably, the activity of Sirt1 in *ApoE* ^KO^; *Larp7* ^tetO^ mice was significantly increased compared with *ApoE* ^KO^; WT mice (*p* = 0.0146, Figure 2D). Together, these results indicated the successful overexpression of Larp7 and the subsequent booster of downstream Sirt1 in *ApoE* ^KO^; *Larp7* ^tetO^ mice.

### 3.3 Activation of Larp7‐Sirt1 axis reduced p53/p65‐mediated cellular senescence signals

**FIGURE 3:**
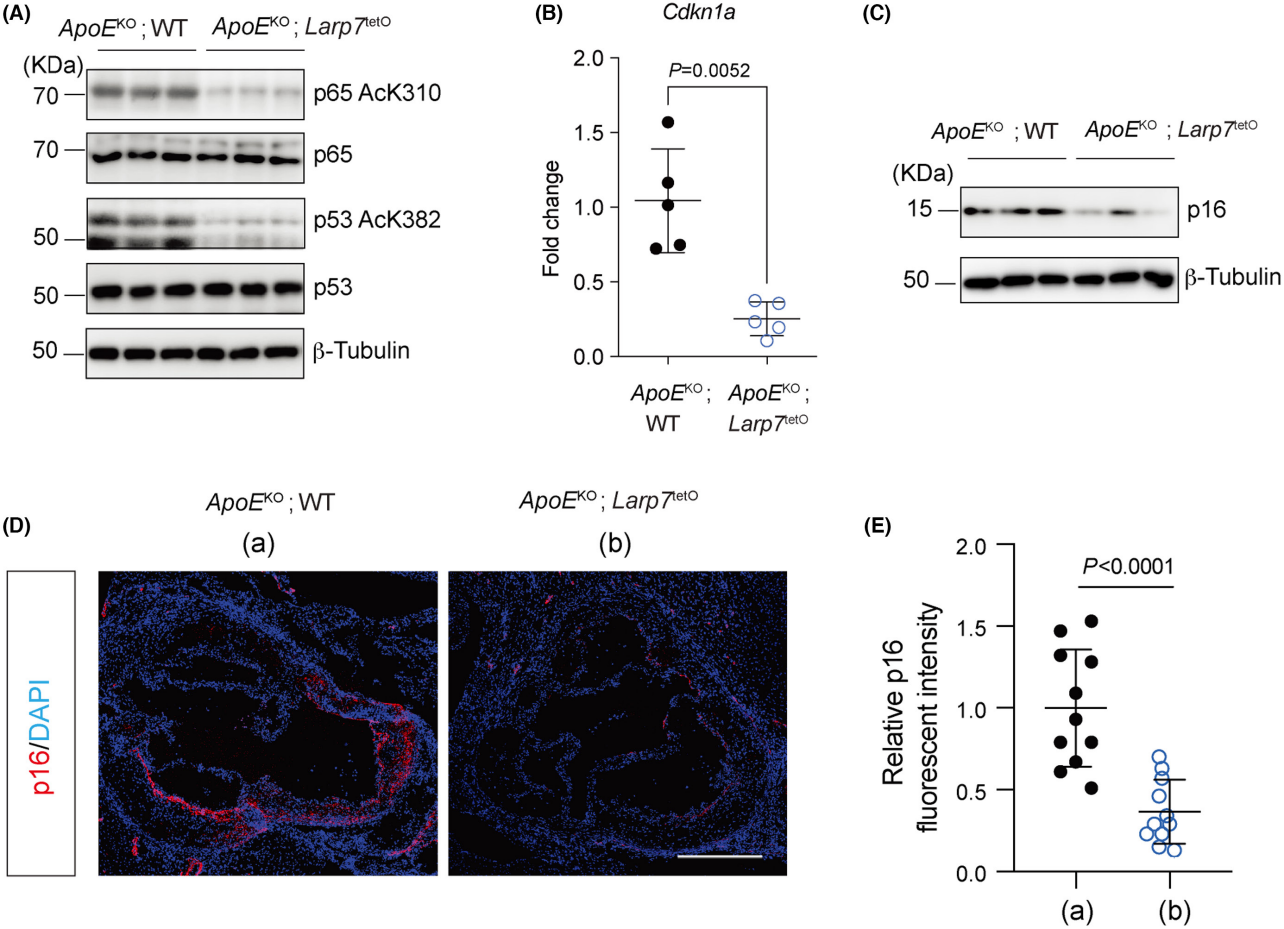
Larp7 overexpression reduced p53/p65 mediated senescent signals in the aortas. (A) Aortas of *ApoE* ^KO^; *Larp7* ^tetO^ mice showing elevated acetylation of p65 and p53 after overexpression of Larp7 for 12 weeks. (B) *Cdkn1a* (p21) mRNA was decreased in *ApoE* ^KO^; *Larp7* ^tetO^ aortas 12 weeks after HFD. *n* = 5 mice per group. Data = Mean ± SD, two‐tailed student's *t*‐test. *p* < 0.05 indicated significance. (C) Immunoblotting showed that p16 protein decreased in *ApoE* ^KO^; *Larp7* ^tetO^ aortas compared with *ApoE* ^KO^; WT aortas. (D) p16 immunostaining showed Larp7 overexpression decreased the senescent cells in the atherosclerotic lesion of *ApoE* ^KO^; *Larp7* ^tetO^ mice compared with *ApoE* ^KO^; WT mice. Scale bar: 500 μm. (a) indicated *ApoE* ^KO^; WT group, (b) indicated *ApoE* ^KO^; *Larp7* ^tetO^ group. (E) p16 positive fluorescent intensity was calculated. *n* = 11 mice per group. Data = Mean ± SD, two‐tailed student's *t*‐test. *p* < 0.05 indicated significance.

Previous studies have established a Larp7‐Sirt1‐p53/p65‐mediated cellular senescence pathway in atherosclerosis.^21^ Sirt1 plays a crucial role in regulating the acetylation of p53 and p65 downstream of Larp7. Acetylation of p65 on Lys310 (p65‐K310Ac) and p53 on Lys382 (p53‐K382Ac) has been shown to enhance the transcriptional activity of NF‐κB and the p53 pathway,^25^, ^26^, ^27^ accounting for the cellular senescence induced by Sirt1 decrease or Larp7 depletion.^21^ To illustrate the direct effects of Larp7 overexpression on this senescence pathway, we first examined the expression levels of p65‐K310Ac, total p65 protein, p53‐K382Ac and total p53 protein. Western blotting results revealed that the expression levels of total p53 and p63 were not affected by Larp7 overexpression. However, the expression levels of p65‐K310Ac and p53‐K382Ac were significantly decreased in *ApoE* ^KO^; *Larp7* ^tetO^ mice compared with *ApoE* ^KO^; WT mice (Figure 3A). These findings indicated that activation of the Larp7‐Sirt1 axis improved the deacetylation of p65 and p53, thereby attenuating their transcriptional activity.

We then proceeded to examine the downstream senescence targets of p65 and p53. One such target is p21^Cip1/Waf1^, also known as Cdkn1a, which is not only a direct target of p53 but also a key marker of cellular senescence. Real‐time quantitative PCR (RT‐qPCR) analysis revealed a downregulation of *Cdkn1a* mRNA levels in *ApoE* ^KO^; *Larp7* ^tetO^ mice compared with *ApoE* ^KO^; WT mice (*p* = 0.0052, Figure 3B). Another reliable marker of senescene, p16, which is an indirect target of p65 and p53, was also found to be decreased in *ApoE* ^KO^; *Larp7* ^tetO^ mice compared with *ApoE* ^KO^; WT mice (Figure 3C).

To further validate these findings, we performed p16 immunostaining at the aortic roots and quantified the average fluorescent intensity of p16. The fluorescence signals of p16 were dramatically reduced in the aortic lesions of Larp7 overexpressed mice compared with WT mice (Figure 3D). The fluorescent intensity of p16 in *ApoE* ^KO^; *Larp7* ^tetO^ mice was reduced to ~15.6% of that in *ApoE* ^KO^; WT mice (*p* < 0.0001, Figure 3E). These results strongly indicated that activation of the Larp7‐Sirt1 axis led to a reduction in cellular senescence mediated by the p65 and p53 signalling pathway.

### 3.4 Larp7 overexpression reduced inflammatory physiologies in atherosclerosis

**FIGURE 4:**
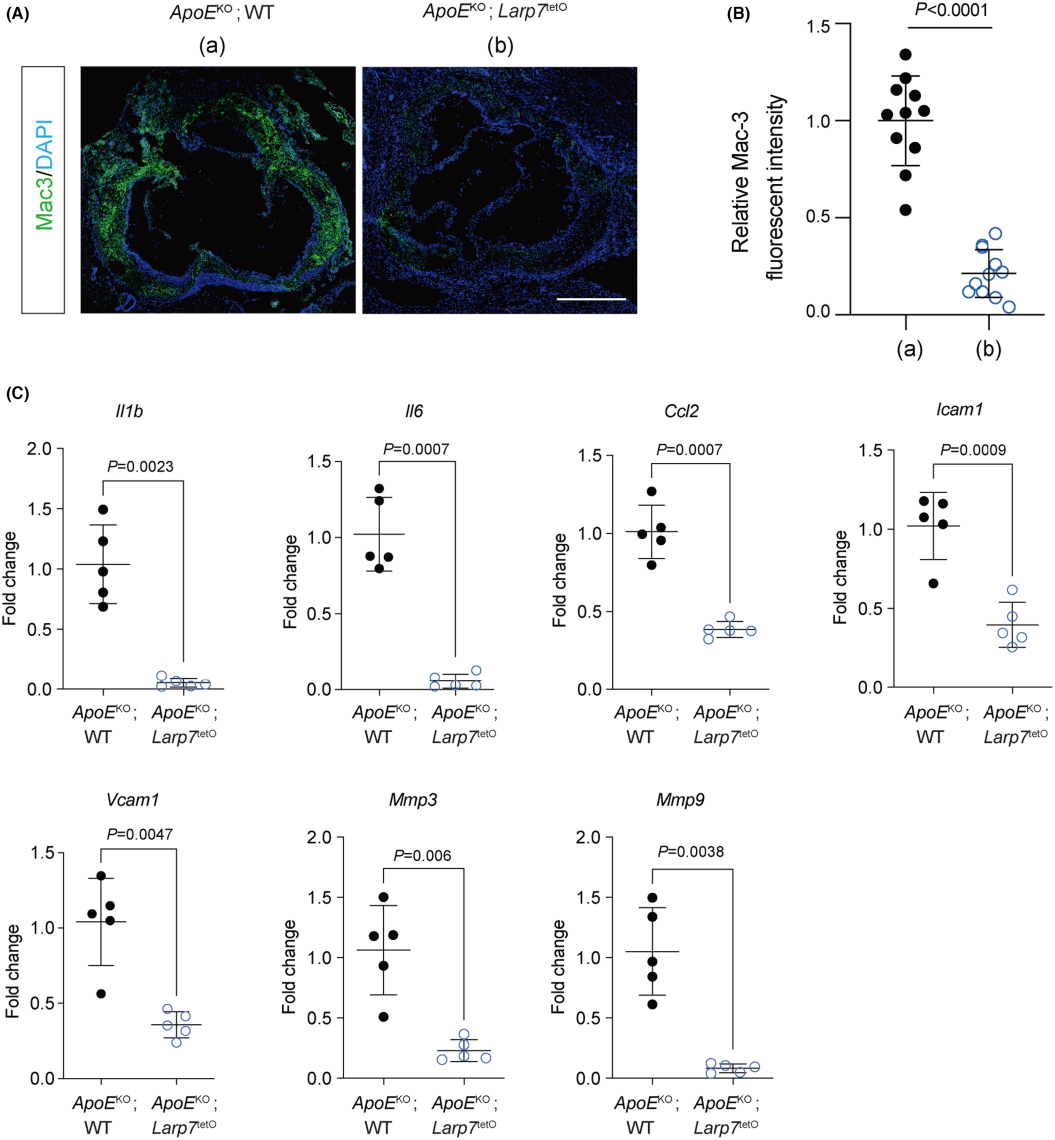
Larp7 overexpression alleviates inflammatory phenotypes in the aortas. (A) Immunostaining of Mac3 in the aortic roots showed Larp7 overexpression decreased the inflammatory macrophage infiltration in the *ApoE* ^KO^; *Larp7* ^tetO^ aortas compared with *ApoE* ^KO^; WT aortas. Scale bar: 500 μm. (a) indicated *ApoE* ^KO^; WT group, (b) indicated *ApoE* ^KO^; *Larp7* ^tetO^ group. (B) Fluorescent intensity of Mac3 was quantified by imageJ. *n* = 11 mice per group. Data = Mean ± SD, two‐tailed student's *t*‐test. *p* < 0.05 indicated significance. (C) RT‐PCR results showed SASP factors were decreased in the *ApoE* ^KO^; *Larp7* ^tetO^ aortas compared with *ApoE* ^KO^; WT aortas. *n* = 5 mice per group. Data = Mean ± SD, two‐tailed student's *t*‐test. *p* < 0.05 indicated significance.

Cellular senescence‐induced inflammatory microenvironment is a major risk factor for atherosclerosis.^2^ To further explore the inhibition effect of Larp7 overexpression on senescence‐mediated pro‐inflammatory phenotype, we performed immunostaining of Mac3 to examine macrophage infiltration in the aortic lesions. The average fluorescent intensity of Mac3 was significantly minimized in the aortic lesion of *ApoE* ^KO^; *Larp7* ^tetO^ mice (Figure 4A). The relative intensity of mac3 in *ApoE* ^KO^; *Larp7* ^tetO^ mice was only 23.3% of that in *ApoE* ^KO^; WT mice (Figure 4B). This reduction in inflammatory macrophage infiltration is consistent with the decrease in inflammatory SASP factors. RT‐PCR analysis found that the expression of SASP factors, including pro‐inflammatory cytokines (*Il1b*, *Il6*, *Ccl2*), metalloproteinases (*Mmp3*, *Mmp9*) and adhesive molecules (Icam1, Vcam1) was almost completely abolished in *ApoE* ^KO^; *Larp7* ^tetO^ mice (Figure 2C). These results clearly indicated that Larp7 overexpression effectively inhibited the inflammatory phenotypes associated with atherosclerosis.

### 3.5 Larp7 overexpression reduced aortic lesion in atherosclerosis

**FIGURE 5:**
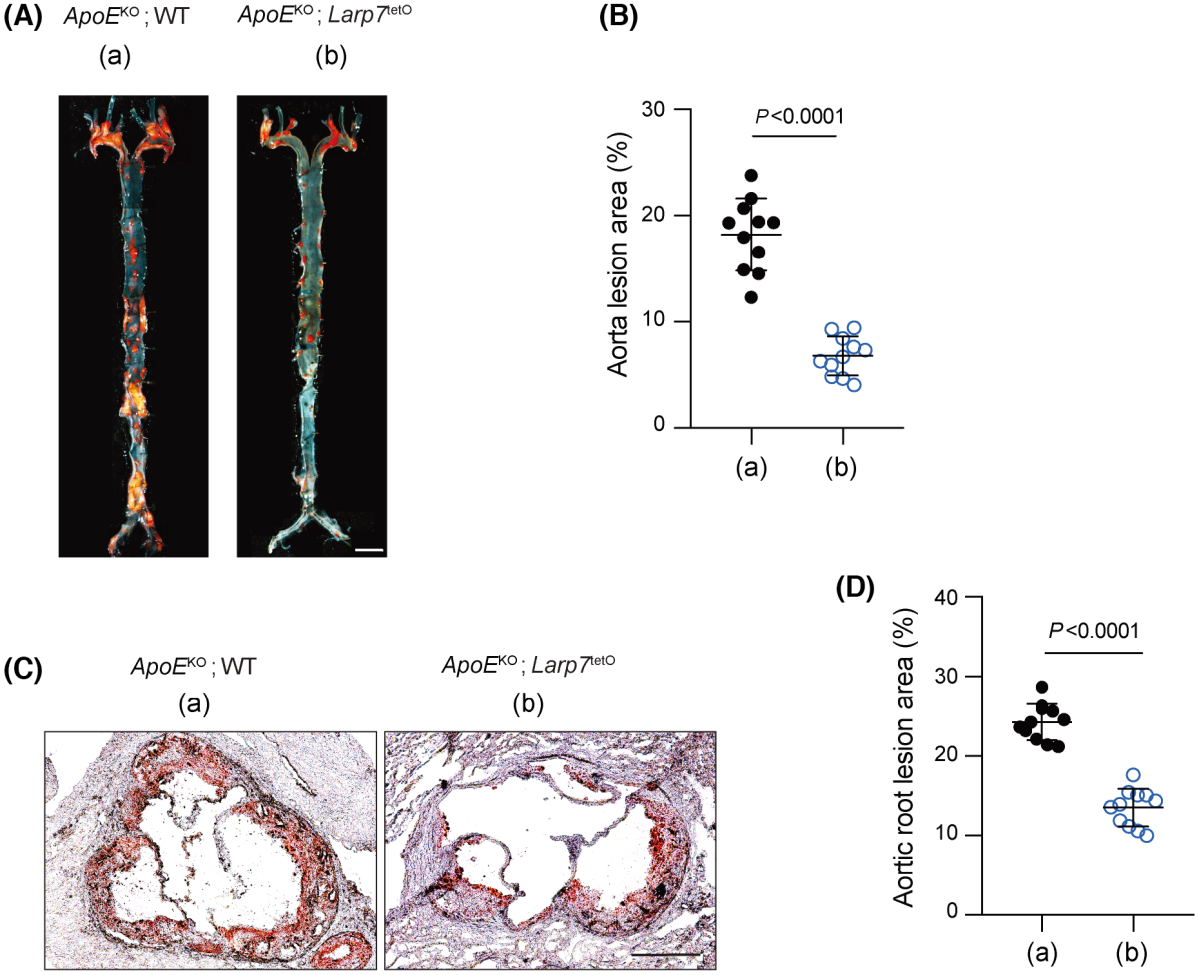
Larp7 overexpression reduced aortic lesion area. (A, B) Larp7 overexpression decreased the atherogenesis. The representative images of atherosclerosis lesions of aorta *en face* stained by oil red O (A). The calculation of percentage of lesion area relative to total region (B). (a) indicated *ApoE* ^KO^; WT group, (b) indicated *ApoE* ^KO^; *Larp7* ^tetO^ group. Scale bar: 1 mm. *n* = 11 mice per group. (C, D) Oil red staining of aortic root. The aortic roots of *ApoE* ^KO^;WT and *ApoE* ^KO^; *Larp7* ^tetO^ mice were cryosectioned and subjected to oil red staining (C), and the relative lesion area (%) was calculated and statistically analysed (D). (a) indicated *ApoE* ^KO^; WT group, (b) indicated *ApoE* ^KO^; *Larp7* ^tetO^ group. Scale bar: 500 μm. *n* = 11 mice per group. Data = Mean ± SD, two‐tailed student's *t*‐test. *p* < 0.05 indicated significance.

The reduction in cellular senescence and inflammation in *ApoE* ^KO^; *Larp7* ^tetO^ mice might result in an alleviation of atherosclerosis. To demonstrate the protective role of Larp7 overexpression in atherosclerosis, we performed Oil red O staining to examine lipid‐laden plaques in atherosclerotic lesions. From the en face view of aorta, Oil red O staining revealed *ApoE* ^KO^; WT mice had numerous lipid plaques, indicated by red colour (Figure 5A). In contrast, the accumulation of lipids was significantly reduced in *ApoE* ^KO^; *Larp7* ^tetO^ mice. The atherosclerotic lesion area of en face aorta in *ApoE* ^KO^; *Larp7* ^tetO^ mice was reduced to approximately one‐third of that in *ApoE* ^KO^; WT mice (*ApoE* ^KO^; *Larp7* ^tetO^ vs. *ApoE* ^KO^; WT mice: 18.2 ± 3.4% vs. 6.8 ± 1.8% ApoE^KO^; *p* < 0.0001).

We further examined lipid plaques in the aortic root. Oil red O staining in the aortic roots also revealed a noticeable decrease in lipids intensity in *ApoE* ^KO^; *Larp7* ^tetO^ mice compared with *ApoE* ^KO^; WT mice. Quantification results indicated that the lesion area in the aortic roots of *ApoE* ^KO^; *Larp7* ^tetO^ mice was reduced to approximately half (*ApoE* ^KO^; *Larp7* ^tetO^ vs. *ApoE* ^KO^; WT mice: 24.3 ± 2.3% vs. 13.5 ± 2.4%, *p* < 0.0001) of that in *ApoE* ^KO^; WT mice (Figure 5D). These findings provide strong evidence that Larp7 overexpression plays a significant role in reducing atherosclerotic plaque formation.

Furthermore, the plasmatic cholesterol and triglyceride levels were not changed in *ApoE* ^KO^; *Larp7* ^tetO^ mice compared with *ApoE* ^KO^; WT mice (Table S1). These results indicated that the atherogenic alleviation in *ApoE* ^KO^; *Larp7* ^tetO^ mice was mainly attributed to the reduction in senescence cells (Figure 3) and the decrease in pro‐inflammatory SASP factors (Figures 2 and 4) rather to an improved lipid metabolism.

**FIGURE 6:**
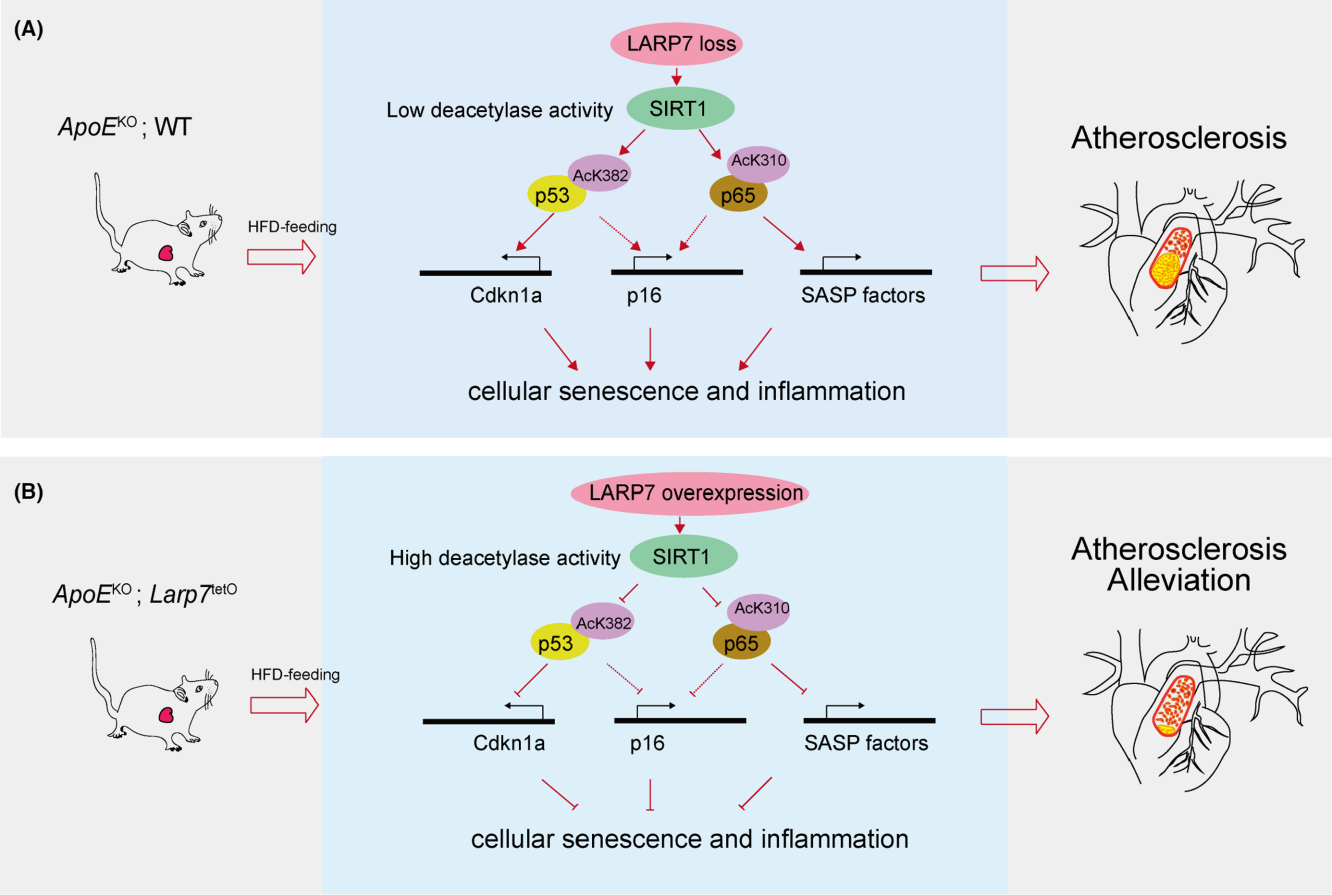
Research summary. (A) HFD intake in *ApoE* ^KO^; WT mice reduces LARP7 protein, lowering SIRT1 activity and increasing p53 and p65 acetylation. This enhances transcriptional activity of *Cdkn1a*, *p16* and SASP factors, leading to cellular senescence and inflammation, promoting atherosclerosis. (B) LARP7 overexpression in *ApoE* ^KO^; *Larp7* ^tetO^ mice restores SIRT1 activity and decreases p53 and p65 acetylation. This inhibits expression of *Cdkn1a*, *p16* and SASP factors, leading to reduced cellular senescence and inflammation, promoting atherosclerosis alleviation.

In sum, our study supports a conclusion that restoring Larp7 expression in hyperlipidaemic mice alleviates aortic senescence and atherogenesis (Figure 6).

## 4 DISCUSSION

Cellular senescence is a key factor for the development of atherosclerosis. Our previous study proved that Larp7 is a novel activator of Sirt1 deacetylase and functions as a gatekeeper of DDR‐induced cellular senescence.^21^ DNA damage activates ATM thus reduces Larp7 protein level and dampens Sirt1 deacetylase activity, increases p53 and p65 acetylation and their transcriptional activity, thereby promoting cellular senescence. The previous study unveiled a novel pathway in the progression of atherosclerosis. But we did not provide a direct proof for the protective role of Larp7 overexpression, which is important to explore a new approach for anti‐atherosclerosis therapy. Here, by generating an inducible Larp7 overexpression hyperlipidaemic (*ApoE* ^KO^; *Larp7* ^tetO^) mouse model, we demonstrate that Larp7 overexpression activates Sirt1 and significantly halts p53/p65 acetylation, and ultimately, ameliorates the vascular senescence and atherogenesis.

Atherosclerosis is a chronic inflammatory disease that leads to arterial atherosclerotic plaques and serious clinical complications like heart attack and stroke.^2^ Risk factors include age, hypercholesterolaemia, smoking, hypertension and diabetes. Sirt1 plays an important role in modulating these risk factors and directly affects atherogenesis and plaque stability by regulating cell proliferation, cell senescence, DNA damage, oxidative stress, cellular metabolism and inflammation in lesions.^28^ Sirt1 reduced in atherosclerotic plaques and results in cell apoptosis, senescence and inflammation of endothelial cells, vascular smooth muscle cells (VSMC) and macrophages.^7^, ^17^, ^18^ Overexpression of sirt1 in these cells both reduces atherosclerosis phenotypes including aortic stiffness, macrophage foam cell formation and inflammation.

Small molecules that activate SIRT1 have shown health benefits in animals since the first description in 2003.^29^ Resveratrol, the most widely studied natural Sirt1 activator, extends the lifespan of HFD‐fed mice and reduces atherosclerosis formation in *ApoE* ^KO^ mice.^29^, ^30^, ^31^, ^32^, ^33^, ^34^ Highly specific Sirt1 activator, like SRT2104, was associated with a moderate improvement in arterial stiffness in type 2 diabetes.^35^ Although these exogenous SIRT1 activators have exhibited promising therapeutic effects on ageing‐related disease in pre‐clinical studies, the endogenous SIRT1 activators are less reported. AROS, the first identified endogenous Sirt1 activator, mediated deacetylation of p53 in a cell‐context‐dependent manner and did not show a correlation of cellular senescence.^36^ While our previous study illustrated Larp7 is possibly a more general and robust activator of Sirt1,^21^ Larp7 protein decreased in ageing and in multiple ageing organs, which is parallel to reduced activity of Sirt1. Larp7 allosteric activates Sirt1 at the N‐terminal domain, a shared mechanism of Sirt1 activators, and did not activate Sirt2, Sirt6 and Sirt7. LARP7 depletion or suppression of Larp7‐Sirt 1 axis exacerbated cellular senescence and atherosclerosis. And Larp7 restoration in this study inhibited atherosclerosis development. Together, these studies demonstrate Larp7 is a specific activator of Sirt1 and is an important partner of Sirt1 in ageing and ageing‐related diseases.

Our study also suggested a translational potential of Larp7 overexpression in preventing ageing and cardiovascular diseases. Unlike systemic administration of chemical Sirt1 activators, which could induce unwanted tumorigenic effects,^37^, ^38^, ^39^, ^40^ Larp7 could achieve tissue‐specific or cellular‐specific overexpression by cellular‐specific promoters. For example, cardiomyocyte‐specific expression of Larp7 protects against heart failure by activating Sirt1‐Pgc1a‐mitochondrial pathway.^22^ Although systemic overexpression of Larp7 in adult mice did not present with obvious detrimental side effects, tissue‐specific overexpression of Larp7 in aortas or in vascular system in future study would give a direct translational guidance of this approach.

Although in this study, we did not measure aortic stiffness in mice, a parameter previously reported to be altered in *ApoE* ^KO^ mice.^41^, ^42^ We speculate that the reduced inflammation and aortic lesions (major contributors to aortic stiffness) in *ApoE* ^KO^; *Larp7* ^tetO^ mice may lead to an alleviation of aortic stiffness, which requires echocardiography analysis in future investigation.

In sum, our study directly proved Larp7 overexpression activates Sirt1 and suppresses cellular senescence pathway, thereby exhibiting a therapeutic role in atherosclerosis.

## AUTHOR CONTRIBUTIONS

**Ping Yang:** Data curation (lead); funding acquisition (equal); investigation (lead); project administration (lead); writing – original draft (lead); writing – review and editing (equal). **Shuo Wu:** Project administration (supporting). **Yige Li:** Project administration (supporting). **Yingmei Lou:** Project administration (supporting). **Junhao Xiong:** Project administration (supporting). **Yuze Wang:** Writing – review and editing (supporting). **Zilong Geng:** Writing – review and editing (supporting). **Bing Zhang:** Conceptualization (lead); funding acquisition (lead); supervision (lead); writing – review and editing (lead).

## CONFLICT OF INTEREST STATEMENT

The authors confirm that there are no conflicts of interest.

## Supporting information


Table S1.

## Data Availability

The data that support the findings of this study are available from the corresponding author upon reasonable request.
