# Supplementary material for: LARP7 overexpression alleviates aortic senescence and atherosclerosis
Source: J Cell Mol Med. 2024 May 31;28(11):e18388. doi: 10.1111/jcmm.18388 (PMC11140237; doi:10.1111/jcmm.18388)
Supplement: Supplementary file 1 — Table S1. [file JCMM-28-e18388-s001.docx]

Supplementary Table 1

The overexpression of LARP7 had no significant effect on blood lipid levels.

|  | ApoE KO ; WT | ApoE KO ; Larp7 TetO | *P* value |
| --- | --- | --- | --- |
| Initial BW (g) | 23.3 ± 3.7 | 23.4 ± 3.2 | 0.9466 |
| Final BW (g) | 34.3 ± 5.9 | 33.3 ± 4.0 | 0.6468 |
| Cholesterol (mg/dl) | 751.9 ± 179.2 | 728.7 ± 287.0 | 0.8224 |
| Triglyceride (mg/dl) | 143.3 ± 48.5 | 126.6 ± 71.2 | 0.5276 |
| n=11 mice in each group. Data= Mean ± SD, two-tailed student’s t test. P<0.05 indicated significance. | | | |
